# Characteristics of patients with coexisting IgA nephropathy and membranous nephropathy

**DOI:** 10.1080/0886022X.2018.1455591

**Published:** 2018-04-05

**Authors:** Pei Chen, Su-Fang Shi, Zhen Qu, Na Zhao, Xin-Fang Xie, Ji-Cheng Lv, Li-Jun Liu, Hong Zhang

**Affiliations:** a Department of Medicine, Renal Division, Peking University First Hospital, Beijing, China;; b Peking University Institute of Nephrology, Beijing, China;; c Key Laboratory of Renal Disease, Ministry of Health of China, Beijing, China;; d Ministry of Education, Key Laboratory of Chronic Kidney Disease Prevention and Treatment (Peking University), Beijing, China

**Keywords:** Anti-PLA_2_R, Gd-IgA1, IgA nephropathy, IgAN–MN, membranous nephropathy

## Abstract

**Background:** Coexistence of IgA nephropathy (IgAN) and membranous nephropathy (MN) in the same patient is rare. Few studies have reported the clinical and pathological features of patients with combined IgAN and MN (IgAN–MN).

**Methods:** The clinico-pathological features, levels of galactose-deficient IgA1 (Gd-IgA1) and autoantibodies against M-type transmembrane phospholipase A_2_ receptor (anti-PLA_2_R) in sera were compared among IgAN–MN, IgAN, and MN patients.

**Results:** Twenty-six patients with biopsy-proven IgAN–MN were enrolled. The mean age at biopsy was 43.6 ± 15.9 years, and 65.4% were male. Proteinuria and estimated glomerular filtration rate (eGFR) levels in patients with IgAN–MN were similar to that of MN patients. Compared with the IgAN patients, IgAN–MN patients showed a higher median proteinuria level (4.3 vs. 1.2 g/day, *p* < .001), and a higher mean eGFR level (101.8 ± 25.4 vs. 78.6 ± 26.9 mL/min/1.73 m^2^, *p* < .001). IgAN–MN patients presented with milder pathological lesions than IgAN patients according to the Oxford Classification. IgAN–MN patients had comparable serum levels of Gd-IgA1 with those of IgAN patients (353.4 ± 95.5 vs. 347.0 ± 109.6 U/mL, *p* = .801). Percentage of IgAN–MN patients with detectable serum levels of anti-PLA_2_R was lower than that of MN patients (38.5% vs. 68.6%, *p* = .011).

**Conclusions:** IgAN–MN patients display similar clinical features to MN patients and milder pathological lesions than IgAN patients. IgAN–MN patients have similar levels of Gd-IgA1 to those of IgAN patients, and a lower proportion of anti-PLA_2_R than MN patients.

## Introduction

IgA nephropathy (IgAN) is the most prevalent primary glomerular disease worldwide [1]. IgAN is characterized by IgA deposition in the glomerular mesangium and extremely variable clinical presentations. The Oxford Classification can be used to predict the risk of progression of IgAN reliably [2]. Multiple studies have established the contribution of aberrantly glycosylated IgA1 in the pathogenesis of IgAN [3–5]. Patients with IgAN have increased serum levels of galactose-deficient IgA1 (Gd-IgA1) [6].

Membranous nephropathy (MN) is a common cause of nephrotic syndrome in adults. MN is characterized by thickening of the glomerular basement membrane, due to the presence of subepithelial immune deposits. Identification of circulating autoantibodies against the M-type transmembrane phospholipase A_2_ receptor (anti-PLA_2_R) is considered to be a promising serologic diagnostic biomarker for idiopathic MN [7,8].

There are few reports [9–16] of the concurrence of IgAN and MN (IgAN–MN). Kobayashi et al. [12] considered that IgAN–MN should be regarded as an entity in glomerular pathology. Stokes and Alpers [17] hypothesized that IgAN–MN results from diverse pathogenetic pathways. However, whether IgAN–MN represents a pathological clinical entity or overlapping of two patterns of kidney injury is not known.

The main aim of the present study was to gain a deeper understanding of patients with IgAN–MN. We studied the clinical manifestations, pathological features as well as levels of Gd-IgA1, anti-PLA_2_R in 26 IgAN–MN patients and compared them with IgAN patients and MN patients.

## Methods

**Figure 1.**
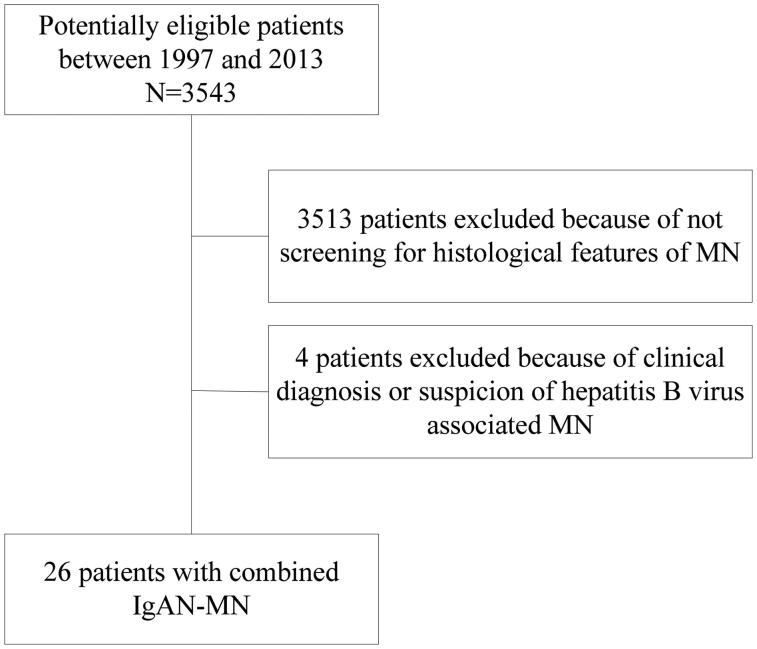
Flowchart of patient selection.

We screened the clinical and pathological data of 3543 patients with IgAN identified from renal biopsies at the Renal Division of Peking University First Hospital (Beijing, China) from January 1997 to December 2013. The diagnosis of IgAN was based on: (i) dominant staining for IgA in the glomerular mesangium on immunofluorescence microscopy; (ii) electron-dense deposits in the mesangium on electron microscopy. Thirty out of 3543 IgAN patients with coexistence of IgG, C3 deposition in glomerular capillary walls and subepithelial electron-dense deposits were diagnosed as having IgAN–MN. Patients with a clinical diagnosis or suspicion of secondary MN (four cases) were excluded from our study. Details of the recruitment process are shown in Figure 1.

### Clinical data

Clinical data submitted at the time of renal biopsy were: serum creatinine level; proteinuria; gross hematuria; hypertension; serum complement (C3,C4) levels and antinuclear antibody (ANA); serum markers of the hepatitis B virus (HBV) and hepatitis C virus (HCV); any other significant clinical/laboratory data. Estimated glomerular filtration rate (eGFR) was calculated using the Chronic Kidney Disease-Epidemiology Collaboration two-level race equation [18]. Follow-up data comprised general clinical course, intervening therapy, and the results of renal function and urinalyses.

All renal biopsies were processed for microscopy (light, electron, immunofluorescence). The proportion of glomeruli with cellular, fibrocellular, or fibrous crescents was recorded. Renal biopsies were scored according to the Oxford Classification in IgAN patients [19,20].

### Gd-deficient IgA1

Gd-IgA1 was detected by a lectin enzyme-linked immunosorbent assay, as reported previously [21]. Briefly, F(ab′)_2_ fragments of goat anti-human IgA (Jackson ImmunoResearch Laboratories, West Grove, PA) were coated onto high-binding MaxiSorp 96-well plates (Nalge-Nunc, Rochester, NY) at 4 °C overnight. After blockade with 1% bovine serum albumin (Sigma-Aldrich, St. Louis, MO), twofold dilutions (1:2000 to 1:16,000) of serum samples and standards were added and incubated overnight at room temperature. A polymeric Gd-IgA1 protein isolated from a patient with multiple myeloma was used as a standard. To remove terminal sialic acid, neuraminidase (Roche Diagnostics, Indianapolis, IN) at 1 mU per well was incubated for 3 h at 37 °C. Terminal N-acetylgalactosamine was detected by biotin-labeled lectin from Helix aspersa (HAA, Sigma-Aldrich, St. Louis, MO) followed by horseradish peroxidase-ExtrAvidin^®^ (Sigma-Aldrich, St. Louis, MO). Absorbance at 490 nm was measured after the reaction had been stopped with 1 mol/L sulfuric acid. The amount of Gd-IgA1 in each sample was calculated using DeltaSoft II (BioMetallics, Princeton, NJ) by interpolating the absorbance on calibration curves constructed using standard Gd-IgA1 myeloma protein. Results were expressed in units per milliliter, whereby 1 unit of Gd-IgA1 was defined as 1 mg standard Gd-IgA1 myeloma protein.

### Anti-PLA_2_R

Circulating anti-PLA_2_R in serum was assessed by a direct immunofluorescence assay (IFA) using HEK293 cells. For transient transfection of HEK293 cells, a complementary DNA encoding full-length PLA_2_R isoform 1 (FA1254-1005-50; Euroimmun, Lübeck, Germany) was used. Detection was performed on an IFA mosaic slide following standard instructions, as established previously [22,23]. Positivity of anti-PLA_2_R was defined as positive staining at serum dilutions ≥1/10. Negativity of anti-PLA_2_R was defined as absence of detectable antibodies at 1/10 dilution.

### Control groups

We searched our follow-up database (which contained clinical, histologic and follow-up data of patients with biopsy-proven IgAN or idiopathic MN from 1997 to 2014) for control-group cases. Fifty-two IgAN patients and 52 MN patients at a ratio of 1:2 were selected by random sampling as two control groups.

### Statistical analyses

Data with normal distributions were presented as the mean ± standard deviation and data with a non-normal distribution as median and quartile values. For comparison of clinical and pathological parameters among different groups, variables with a normal distribution were analyzed by Student’s *t*-test (two groups) and non-parametric variables were analyzed by the Mann–Whitney test or Kruskal–Wallis test. Differences in proportions were tested by the chi-square test. Statistical analyses were undertaken using SPSS version 19.0 (IBM, Armonk, NY). *p* < .05 was considered statistically significant.

## Results

### Clinical data

Clinical data are summarized in Table 1. There were 17 (65.4%) males and nine (34.6%) females among patients with IgAN–MN. IgAN–MN patients were older than IgAN patients (43.6 ± 15.9 vs. 34.9 ± 11.1 years, *p* = .017), but younger than MN patients (43.6 ± 15.9 vs. 51.4 ± 13.3 years, *p* = .026). Prevalence of nephrotic syndrome, proteinuria and eGFR levels in patients with IgAN–MN were similar to that of MN patients. Compared with the IgAN patients, IgAN–MN patients showed a greater prevalence of nephrotic syndrome (61.5% vs. 0.0%, *p* < .001), a higher median proteinuria level (4.3 vs. 1.2 g/day, *p* < .001), and a higher mean eGFR level (101.8 ± 25.4 vs. 78.6 ± 26.9 mL/min/1.73 m^2^, *p* < .001). There were no significant differences in the prevalence of hypertension between patients with IgAN–MN and IgAN patients (26.9% vs. 34.6%, *p* = .493) or MN patients (26.9% vs. 28.8%, *p* = .859).

**Table 1.** Clinical characteristics of IgAN–MN patients, IgAN patients and MN patients.

Characteristics	IgA–MN	IgAN	MN	*p* (IgAN–MN vs. IgAN)	*p* (IgAN–MN vs. MN)
Number of patients	26	52	52		
Baseline characteristics					
Gender (male)	17 (65.4)	25 (48.1)	30 (57.7)	.148	.513
Age (years)	43.6 ± 15.9	34.9 ± 11.1	51.4 ± 13.3	.017	.026
Gross hematuria	3 (11.5)	16 (30.7)	0 (0)	.056	.061
Hypertension	7 (26.9)	18 (34.6)	15 (28.8)	.493	.859
Initial proteinuria (g/day)	4.3 (2.4, 6.3)	1.2 (0.7, 1.9)	5.1 (3.1, 7.1)	<.001	.455
eGFR (mL/min/1.73 m^2^)	101.8 ± 25.4	78.5 ± 26.9	100.2 ± 21.6	<.001	.778
Nephrotic syndrome	16 (61.5)	32 (61.5)	33 (63.5)	<.001	.868
Therapy					
ACE inhibitors or ARBs	16 (61.5)	51 (98.1)	29 (55.8)	<.001	.627
Glucocorticoids	15 (57.7)	26 (50.0)	38 (73.1)	.521	.170
Any other immunosuppressive agents	11 (42.3)	16 (30.8)	38 (73.1)	.313	.008
Follow-up^a^					
Follow-up interval (months)	22.8 (10.1, 81.4)	82.3 (47.8, 114.7)	37.4 (24.6, 69.6)	<.001	.053
Kidney progression event^b^	2 (10.5)	8 (15.4)	3 (6.1)	.892	.865
Partial remission and complete remission^c^	14 (73.7)	–	39 (75.0)	–	.845

eGFR: estimated glomerular filtration rate.

Values were presented as *n* (%) for number (%), mean ± standard for continuous, median (25–75% interquartile) for non-normally distributed continuous variables.

^a^Follow-up data were available only in 19 of the 26 patients with combined IgAN–MN.

^b^Kidney progression event was defined as eGFR decreasing by half, or end-stage renal disease.

^c^Partial remission was defined as proteinuria <3.5 g/day plus a 50% reduction from its peak value; complete remission was defined as proteinuria <0.3 g/day.

### Pathology

Pathological findings are summarized in Table 2. Most patients with IgAN–MN had diffuse global thickening of capillary walls, and three patients also had formation of silver-positive ‘spikes’. Mesangial expansion and variable mesangial hypercellularity were observed in all patients. Crescent formation was found in four patients, with the percentage ranging from 3.5% to 48.5%.

**Table 2.** Pathological characteristics of IgAN–MN patients and IgAN patients.

Characteristic	IgA–MN	IgAN	*p*
M1	19 (73.1)	38 (67.9)	1.000
E1	5 (19.2)	32 (61.5)	<.001
S1	3 (11.5)	41 (78.8)	<.001
T1	6 (23.1)	10 (19.2)	.223
T2	0 (0.0)	6 (11.5)	
C1	3 (11.5)	18 (34.6)	.007
C2	1 (3.8)	9 (17.3)	

M: mesangial hypercellularity; E: endocapillary hypercellularity; S: segmental glomerulosclerosis; T: tubular atrophy/interstitial fibrosis; C: crescents.

Values were presented as *n* (%) for number (%), median (25–75% interquartile) for non-normally distributed continuous variables.

Data of the Oxford Classification for IgAN–MN and IgAN patients are summarized in Table 1. There were significant differences in the scores of E0/1, S0/1, and C0/1/2 between patients with IgAN–MN and IgAN patients (21/5 vs. 20/32, *p* < .001; 23/3 vs. 11/41, *p* < .001; 22/3/1 vs. 25/18/9, *p* = .007). In summary, patients with IgAN–MN presented with milder pathological lesions than those of IgAN patients according to the Oxford Classification.

### Gd-IgA1

There was no significant difference in serum levels of Gd-IgA1 between patients with IgAN–MN and IgAN patients (353.4 ± 95.5 vs. 347.0 ± 109.6 U/mL, *p* = .801).

### Anti-PLA_2_R

The percentage of IgAN–MN patients who had detectable serum levels of anti-PLA2R was significantly lower than that for MN patients (38.5% vs. 68.6%, *p* = .011).

### Follow-up

Follow-up data were available for 19 patients with IgAN–MN. They were followed up for 4–106 months. Two patients achieved a 50% decline in eGFR or doubling of serum creatinine levels, which was similar to observations in IgAN patients and MN patients (10.5% vs. 15.4%, *p* = .892; 10.5% vs. 6.1%, *p* = .865). Eight patients achieved partial remission (proteinuria <3.5 g/day plus a 50% reduction from its peak value) and six patients achieved complete remission (proteinuria <0.3 g/day). Prevalence of remission (partial remission and complete remission) of patients with IgAN–MN was comparable with that of MN patients (73.7% vs. 75.0%, *p* = .845).

## Discussion

Prevalence of IgAN–MN is relatively low. Reported cases have been characterized by severe proteinuria and stable renal function [17]. Up until now, a well-conducted comparison among such patients is lacking. Our study comprised 26 patients with IgAN–MN, and we compared these patients with IgAN patients and MN patients.

We found that patients with IgA–MN shared some similarities with regard to proteinuria severity, renal function, and prevalence of nephrotic syndrome with MN patients. According to the Oxford Classification, patients with IgAN–MN showed milder pathological lesions than those of IgAN patients. IgAN–MN patients had comparable serum levels of Gd-IgA1 with those of IgAN patients. However, the proportion of anti-PLA_2_R detected in the serum of patients with IgAN–MN was lower than that of patients with primary MN.

Finding a shared pathogenetic pathway leading to the coexistence of IgAN–MN is difficult. Kobayashi et al. [12] considered that the coexistence of IgAN–MN did not occur due to chance alone, and should be regarded as an entity in glomerular pathology. According to our study, definition of IgAN–MN as an atypical subclass of IgAN or MN is not convincing.

Patients with IgAN usually present with gross hematuria accompanied by a prodromal infection of the upper respiratory tract or gastrointestinal system. Three patients with IgAN–MN had a history of gross hematuria, but most patients with IgAN–MN presented only with microscopic hematuria. Nephrotic syndrome was uncommon in IgAN patients, except in those with minimal changes in pathological features. However, 61.5% of patients with IgAN–MN had nephrotic syndrome. These data are in accordance with a study which concluded that patients with IgAN–MN present with severe proteinuria [17]. To conclude, the clinical features of patients with IgAN–MN are not in accordance with those of IgAN patients.

Several studies have established the contribution of Gd-IgA1 in IgAN pathogenesis [3,5], so Gd-IgA1 is regarded as an important biomarker of IgAN. In our study, patients with IgA–MN had comparable levels of Gd-IgA1 to those of IgAN patients, suggesting that Gd-IgA1 might contribute (at least in part) to IgAN–MN pathogenesis.

Clinical features between patients with IgA–MN and MN patients were quite similar, so it is reasonable to hypothesize that IgA–MN might be a type of secondary MN. In two studies [13,14], two patients with HBV infection showed staining for mesangial IgA, subepithelial IgG, and HBV antigens in the glomeruli simultaneously, supporting the notion that HBV antigens have a pathogenetic role in the simultaneous development of IgAN–MN. In our study, five patients with IgA–MN had serologic evidence of previous infection with the HBV, but HBV antigens were not detected by immunofluorescence microscopy in glomeruli. In addition, three patients with IgA–MN in our study had a positive ANA test, and one patient had a positive SMA test, but no patients had other serologic or clinical evidence of autoimmune diseases. Therefore, none of these patients could be diagnosed as having secondary MN in our study.

Frasca et al. [10] reported that IgAN–MN occurred separately in one patient at an interval of 7 years. Miyazaki et al. [15] reported that IgAN developed 14 years after a diagnosis of MN. They postulated that discontinuation of immunosuppressive agents during the course of MN might enhance IgA synthesis and induce IgAN. Those two cases suggested that IgAN–MN could occur in the same patient separately at an interval of several years, indicating that IgA–MN might represent a result of two glomerular diseases coexisting in the same patient. In our study, the onset of each nephropathy was not known because two nephropathies were discovered simultaneously by renal biopsy. However, our research showed that patients with IgAN–MN were older than IgAN patients and younger than MN patients. Onset of IgAN–MN might result from superimposed MN aggravating renal lesions in patients with previous mild IgAN. However, how IgAN predisposes to the onset of MN is unknown. In our study, the histologic lesions of patients with combined IgAN–MN were milder than IgAN patients according to Oxford Classification. We speculated that occurrence of superimposed MN on a background of preexisting mild IgAN caused combined IgAN–MN.

Circulating anti-PLA_2_R has been regarded as an important biomarker to discriminate between idiopathic MN and secondary MN. The significant difference of the proportion of detective serum anti-PLA2R in the two groups indicated that the pathogenetic mechanisms of combined IgAN–MN might be different with idiopathic MN.

Our study has several limitations. First, the follow-up data was incomplete. As many as seven patients with combined IgAN–MN were lost to follow-up. Second, the follow-up intervals of IgAN patients were notably longer than patients with combined IgAN–MN. Third, we did not have the result of subclasses of IgG deposition in renal in patients with combined IgAN–MN.

In conclusion, patients with combined IgAN–MN displayed similar clinical features with MN patients and milder pathological lesions than IgAN patients. The pathogenesis of combined IgAN–MN was different with either IgAN or MN.
